# Successful Treatment of Bacteremia and Ventilator-Associated Pneumonia Caused by KPC/OXA-48-like *Klebsiella pneumoniae* Co-Producer with a Continuous Infusion of High-Dose Meropenem Plus Fosfomycin Guided by Real-Time Therapeutic Drug Monitoring

**DOI:** 10.3390/idr14010010

**Published:** 2022-01-21

**Authors:** Pier Giorgio Cojutti, Giacomo Fornaro, Milo Gatti, Matteo Rinaldi, Paolo Gaibani, Maddalena Giannella, Federico Pea, Pierluigi Viale

**Affiliations:** 1 SSD Clinical Pharmacology, Department for Integrated Infectious Risk Management, IRCCS Azienda Ospedaliero Universitaria di Bologna, 40138 Bologna, Italy; piergiorgio.cojutti@aosp.bo.it (P.G.C.); milo.gatti2@unibo.it (M.G.); 2 Department of Medical and Surgical Sciences, Alma Mater Studiorum, University of Bologna, 40138 Bologna, Italy; giacomo.fornaro@aosp.bo.it (G.F.); matteo.rinaldi@aosp.bo.it (M.R.); maddalena.giannella@unibo.it (M.G.); pierluigi.viale@unibo.it (P.V.); 3 Infectious Diseases Unit, Department for Integrated Infectious Risk Management, IRCCS Azienda Ospedaliero Universitaria di Bologna, 40138 Bologna, Italy; 4 Division of Microbiology, Department for Integrated Infectious Risk Management, IRCCS Azienda Ospedaliero Universitaria di Bologna, 40138 Bologna, Italy; paolo.gaibani@aosp.bo.it

**Keywords:** continuous infusion of meropenem, therapeutic drug monitoring, critically ill patient

## Abstract

Bacteremia and ventilator-associated pneumonia due to a pan-resistant *Klebsiella pneumoniae* strain co-producing KPC and OXA-48 carbapenemases was successfully treated in a COVID-19 critically ill patient with a combination therapy of a high-dose continuous infusion of meropenem (up to 3 g every 6 h, daily) plus fosfomycin (up to 24 g/daily) that was guided by real-time therapeutic drug monitoring. Clinical pharmacological advice was helpful in maximizing, over time, the pharmacodynamic target attainment of both antibiotics.

## 1. Introduction

Carbapenem-resistant Enterobacterales (CRE) have spread globally and are responsible for difficult-to-treat infections with increased morbidity and mortality [1]. *Klebsiella pneumoniae* is the third-most-frequent healthcare-associated pathogen, and the most prevalent one among CRE [2]. The predominant beta-lactamase produced by *Klebsiella pneumoniae* is KPC, but the proportion of OXA-48 is increasing [3]. Treatment options for these infections are quite limited. Combination therapy could be helpful, but multi-drug resistance to several antibiotics, including aminoglycosides, tigecycline, and colistin, often emerged [4]. The recently approved new beta-lactam/beta-lactamase inhibitor combinations ceftazidime/avibactam and meropenem/vaborbactam have improved the opportunities against KPC- and some OXA-48-producing *Klebsiella pneumoniae* [5]. However, antimicrobial selection is still taken on a patient-by-patient basis by considering the microbiological susceptibility and the site of infection, with the need of attaining an adequate pharmacokinetic/pharmacodynamic target of efficacy.

Here, we describe a case of bacteremia and ventilator-associated pneumonia (VAP) due to an extensively drug-resistant (XDR) *Klebsiella pneumoniae* strain resistant to novel beta-lactam/beta-lactamase inhibitors and to cefiderocol that was successfully treated in a critically ill patient with a combination of a high-dose continuous infusion of meropenem plus fosfomycin guided by real-time therapeutic drug monitoring.

## 2. Methods and Results

A 58-year-old critically ill patient with severe SARS-CoV-2 infection (Siddiqi classification IIIb) [6] was admitted to the intensive care unit and required mechanical ventilation. The clinical course was complicated by pulmonary venous thromboembolism and acute kidney injury, such that respiratory support with extracorporeal membrane-oxygenation (ECMO) and renal replacement therapy with continuous venovenous hemodiafiltration (CVVHDF) were needed. At that time, he suffered from a VAP episode caused by *Pseudomonas aeruginosa* and a central venous catheter (CVC)-related candidemia caused by *Candida parapsilosis.* These infections were successfully treated with a two-week combination therapy of a continuous infusion (CI) of meropenem plus colistin and with caspofungin, respectively. Subsequently, ECMO was withdrawn, whereas CVVHDF was maintained for supporting renal function (PrismaFlex filter set (Gambro, Lakewood, CO, USA) with an AN69 high-flux ST-150 membrane and ultrafiltration, blood flow, dialysate fluid, and replacement fluid rates of 2855 mL/h, 150 mL/h, 500 mL/h, and 1000 mL/h, respectively). Measured 24 h creatinine clearance (mCL_CR_) showed a residual renal function of 33.3 mL/min.

Five days after completing antimicrobial treatment, while the patient was afebrile, hemodynamically stable, and still on mechanical ventilation, the inflammatory biomarkers started increasing (C-reactive protein (C-RP), procalcitonin (PCT), and white blood cells raised from 5.6 mg/dL, <0.1 ng/mL, and 5.76 × 10^9^/L to 23 mg/dL, 3.4 ng/mL, and 8.98 × 10^9^/L, respectively). Blood and bronchoalveolar lavage cultures were performed, and from both, XDR *Klebsiella pneumoniae* were yielded. The XDR *Klebsiella pneumoniae* strains isolated from the two sites had the same susceptibility pattern. They were susceptible only to fosfomycin (MIC of 32 mg/L, assessed by agar dilution) and gentamycin (MIC of 2 mg/L), and resistant to meropenem (MIC of 16 mg/L), colistin (32 mg/L), ceftazidime/avibactam (32 mg/L), meropenem/vaborbactam (32 mg/L), and cefiderocol (16 mg/L). Phenotypic and molecular characterization confirmed that both *Klebsiella pneumoniae* strains co-produced KPC and OXA-48 carbapenemases. Whole-genome sequencing was performed for identifying the molecular mechanism of resistance to novel beta-lactams, as previously described [7]. Assembled genomes were screened for known antimicrobial resistance, sequence type (ST), and plasmid content by a CGE server (https://cge.cbs.dtu.dk/services/MLST/ (accessed on 1 October 2021)). Porin genes were manually investigated by BLAST analysis. Genomic analysis showed that both XDR *Klebsiella pneumoniae* belonged to ST512 and shared similar genes encoding antimicrobial resistance. Of note, analysis of carbapenemase genes showed co-harboring of *bla*_KPC-66_ and *bla*_OXA-181_ carbapenemase genes.

Combination therapy with a high-dose CI of fosfomycin (24 g/day, after 4 g loading dose) plus a high-dose CI of meropenem (1.5 g every 6 h over 6 h, after 1 g loading dose) was started [8]. This choice was based on several assumptions. Fosfomycin monotherapy was considered unfeasible due to the high risk of rapid selection of multi-drug-resistant (MDR) Gram-negative strains, as usually recommended [9]. Meropenem was preferred to gentamicin for combination with fosfomycin because on the one hand gentamicin was considered inconvenient according to the patient’s renal failure, and on the other hand the MIC for meropenem of the XDR *Klebsiella pneumoniae* isolates was 16 mg/L, a value permissive for pharmacokinetic/pharmacodynamic (PK/PD) optimization with high-dose CI dosing regimens guided by real-time therapeutic drug monitoring (TDM).

**Figure 1:**
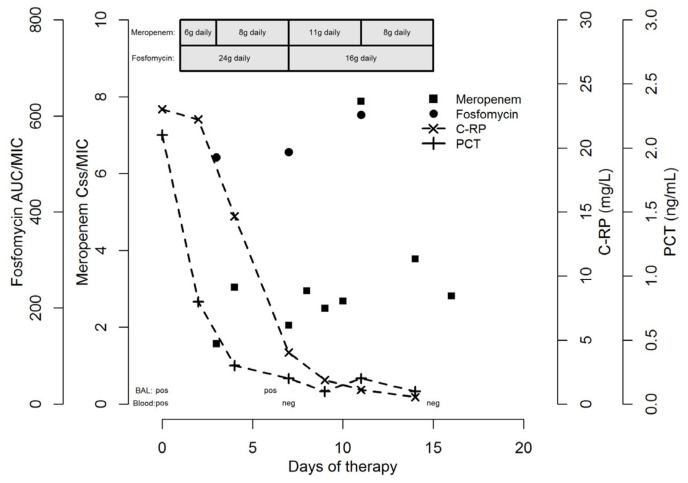
Temporal trends of pharmacokinetic/pharmacodynamic indices of meropenem (Css/MIC) and fosfomycin (AUC_24h_/MIC), as well as a reduction in inflammatory biomarkers (C-reactive protein and procalcitonin). AUC_24h_, 24 h area under the plasma concentration–time curve; Css, steady-state concentration; C-RP, C-reactive protein; PCT, procalcitonin; and MIC, minimum inhibitory concentration.

A real-time clinical pharmacological advice program based on TDM was established to optimize treatment. The desired pharmacodynamic targets were set at a steady-state-plasma-concentration-to-MIC ratio (Css/MIC) of 1–4 for meropenem [10] and at a twenty-four-hour-area-under-the-plasma-concentration–time-curve-to-MIC ratio (AUC_24h_/MIC) of >83 for fosfomycin [11]. At the first TDM assessment (day 2), the Css/MIC ratio of meropenem was borderline (1.57) and a dosage increase to 2 g q6h over 6 h was applied, whereas for fosfomycin the AUC_24h_/MIC ratio was more than optimal (513.7) and the dosing regimen was confirmed. Clinical conditions improved within 72 h from starting therapy (C-RP and PCT dropped to 14.66 mg/L and 0.3 ng/mL, respectively). On day 7 the Css/MIC ratio of meropenem was 2.05, and the dosage was furtherly increased up to 3 g q6h over 6 h with the intent of approximating the Css/MIC ratio to 4. The AUC_24h_/MIC ratio of fosfomycin was still more than optimal (524.5), and the dosage was reduced to 16 g/day CI. After stopping CVVHDF on day 11, the meropenem Css/MIC ratio rose up to 7.88, such that the dosage was reduced to 2 g q6h over 6 h. The meropenem Css/MIC ratio decreased to 3.78 on day 14 and to 2.82 on day 16 (Figure 1).

Fosfomycin dosage was not modified furtherly. Two consecutive negative blood cultures (on day 7 and 15) confirmed the eradication of the XDR *Klebsiella pneumoniae*. The patient was weaned from mechanical ventilation and transferred to a low-intensity medical care unit where he completed antimicrobial treatment up to day 17.

## 3. Discussion

This case offers several aspects of innovation from both a microbiological and pharmacological point of view. To the best of our knowledge, this is the first report of a carbapenem-resistant *Klebsiella pneumoniae* co-producing a KPC variant conferring ceftazidime–avibactam resistance (i.e., KPC-66) and a class D carbapenemase (i.e., OXA-181). So far, *Klebsiella pneumoniae* strains harboring KPC-66 have been identified only in the USA, and isolates co-producing KPC and OXA-48-like carbapenemase are still rare in Italy [12]. We recently yielded some KPC and OXA-48 co-producer *Klebsiella pneumoniae* strains in our hospital, but so far they were all susceptible to ceftazidime/avibactam. It is worth noting that this isolate expressed an XDR profile that included all the novel beta-lactams and/or beta-lactam/beta-lactamase inhibitors (ceftazidime/avibactam, meropenem/vaborbactam, and cefiderocol). In this scenario, the possibility of using a high-dose CI of meropenem as a combination therapy with fosfomycin was made feasible thanks to the availability of a real-time TDM-based clinical pharmacological advice program that allowed the low grade of in vitro resistance to meropenem to be overcome by maximizing treatment optimization. In this regard, it was previously shown that among 30 patients with KPC *Klebsiella pneumoniae* infections (with 53.3% of strains having an MIC of meropenem ≥ 16 mg/L), a TDM-guided high-dose CI of meropenem up to 13.2 g daily focused on targeting the Css/MIC ratio at >1 allowed for a clinical cure rate as high as 73.3% [10]. A CI administration of a high dose of fosfomycin also ensured the attainment of a valuable AUC_24h_/MIC ratio. Our choice of combining meropenem with fosfomycin is in agreement with the findings of a recent case series of seven patients with XDR *Klebsiella pneumoniae* infections who were successfully treated with this combination and in whom synergism between meropenem and fosfomycin against the XDR *Klebsiella pneumoniae* clinical isolates was documented by using time–kill assays [13]. Moreover, it has been reported that this combination may also have a role in killing subpopulations that are heteroresistant to fosfomycin [14].

## 4. Conclusions

In conclusion, a real-time TDM-based clinical pharmacological advice program for optimizing combination therapy with a high-dose CI of meropenem plus fosfomycin could represent a valuable option for the treatment of XDR-resistant KPC- and OXA-48-co-producing *Klebsiella pneumoniae* resistant to cefiderocol and to the new beta-lactam/beta-lactamase inhibitors.

## Data Availability

Not applicable.
